# Src kinase inhibition with dasatinib impairs neutrophil function and clearance of *Escherichia coli* infection in a murine model of acute lung injury

**DOI:** 10.1186/s12950-020-00261-5

**Published:** 2020-10-30

**Authors:** James G. Macfarlane, David A. Dorward, Marie-Hélène Ruchaud-Sparagano, Jonathan Scott, Christopher D. Lucas, Adriano G. Rossi, A. John Simpson

**Affiliations:** 1 Translational and Clinical Research Institute, Newcastle University, Newcastle upon Tyne, NE2 4HH UK; 2 Centre for Inflammation Research, Queen’s Medical Research Institute, University of Edinburgh, Edinburgh, EH16 4TJ UK

**Keywords:** Dasatinib, Src kinases, Neutrophil, Acute lung injury, acute respiratory distress syndrome, Pneumonia, Phagocytosis, Infection

## Abstract

**Background:**

Neutrophils rapidly respond to and clear infection from tissues, but can also induce tissue damage through excessive degranulation, when acute inflammation proceeds unchecked. A number of key neutrophil functions, including adhesion-dependent degranulation, are controlled by src family kinases. Dasatinib is a potent src inhibitor used in treating patients with chronic myeloid leukaemia and treatment-resistant acute lymphoblastic leukaemia. We hypothesized that dasatinib would attenuate acute inflammation by inhibiting neutrophil recruitment, degranulation and endothelial cell injury, without impairing bacterial clearance, in a murine model of bacteria-induced acute lung injury. C57BL/6 mice received intratracheal *Escherichia coli*, and were treated with intraperitoneal dasatinib or control. Bacterial clearance, lung injury, and markers of neutrophil recruitment and degranulation were measured. Separately, human blood neutrophils were exposed to dasatinib or control, and the effects on a range of neutrophil functions assessed.

**Results:**

Dasatinib was associated with a dose-dependent significant increase in *E. coli* in the mouse lung, accompanied by impairment of organ function, reflected in significantly increased protein leak across the alveolar-capillary membrane. However, the number of neutrophils entering the lung was unaffected, suggesting that dasatinib impairs neutrophil function independent of migration. Dasatinib did not cause direct toxicity to human neutrophils, but led to significant reductions in phagocytosis of *E. coli*, adhesion, chemotaxis, generation of superoxide anion and degranulation of primary and secondary granules. However, no biologically important effect of dasatinib on neutrophil degranulation was observed in mice.

**Conclusions:**

Contrary to our starting hypothesis, src kinase inhibition with dasatinib had a detrimental effect on bacterial clearance in the mouse lung and therefore does not represent an attractive therapeutic strategy to treat primary infective lung inflammation. Data from human neutrophils suggest that dasatanib has inhibitory effects on a range of neutrophil functions.

## Background

Neutrophils are key cellular mediators involved in rapidly responding to and clearing bacterial and fungal infection. They are recruited early to the alveoli of patients with pulmonary sepsis, acute lung injury (ALI) and acute respiratory distress syndrome (ARDS) and their levels correlate with degree of hypoxia, protein leak and clinical outcome [1–3]. Early activation is achieved by coordinated retention of circulating neutrophils by endothelial cells, followed by neutrophil extravasation, chemotaxis towards pathogens, and ingestion of these pathogens in discrete phagosomes [4, 5]. Following phagocytosis, neutrophil granules containing proteolytic enzymes and machinery to generate reactive oxygen species fuse with the phagosome, and killing of pathogens proceeds [6–8]. Local, extracellular release of granule products is potently anti-microbial and pro-inflammatory, and can therefore be considered as a physiological response when regulated. However, depending on the size of the inflammatory stimulus and the intensity of the neutrophil response, excessive neutrophil granule release may become pathological, with neutrophil proteases, cytokines and reactive oxygen species (ROS) capable of direct and indirect damage to the alveolar epithelium, leading to increased permeability and protein-rich fluid leak into the alveolar space [9, 10].

Several key neutrophil functions are regulated by src kinases. Adhesion-dependent neutrophil degranulation is regulated by the src kinases Hck and Fgr [11]. Neutrophils from mice deficient in Hck, Fgr and Lyn fail to degranulate or generate superoxide anion in response to the bacterial virulence factor formyl methionine leucine phenylalanine (fMLF) [12]. Activation of neutrophils via cell surface Fc gamma receptors is also impaired in vivo in Hck/Fgr/Lyn-null mice [13].

Dasatinib is a src kinase inhibitor that is widely used in the treatment of Philadelphia chromosome-positive chronic myeloid leukemia (CML) and treatment-resistant acute lymphoblastic leukaemia (ALL), with the potential for use in other malignancies being actively explored [14, 15]. In vitro, dasatinib blocks adhesion-dependent neutrophil degranulation, with little effect on chemotaxis, phagocytosis or bacterial killing at serum concentrations of 50–100 nM, similar to physiological concentrations measured in dasatinib-treated CML patients [16]. In a murine lipopolysaccharide (LPS)-induced model of acute lung injury, dasatinib attenuated lung inflammation but its effect was dependent on the dose and the method of LPS delivery [17]. In the caecal ligation and puncture (CLP) model of sepsis, pre- and post-CLP administration of low dose dasatinib (1 mg/kg) improved survival by 50% whereas survival was worsened by a higher dose (10 mg/kg) [18].

Based on these in vitro and in vivo data, our starting hypothesis was that src kinase inhibition with dasatinib would prove beneficial in infective ALI/ARDS, by attenuating neutrophilic inflammation, while maintaining sufficient neutrophil phagocytic and killing capacity to clear infection. Until now the effect of dasatinib in models of direct bacterial lung infection has been poorly characterized. We therefore sought to test our hypothesis in an in vivo model of bacteria-induced acute lung injury. As described below, our data did not support our starting hypothesis, instead suggesting that dasatinib is associated with reduced bacterial clearance.

## Results

### Dasatanib impairs lung clearance of *E. coli* infection and increases alveolar leak

Experiments were performed to establish the time course of *E. coli*-mediated recruitment of neutrophils to the alveolar space and pulmonary interstitium (see Supplementary Fig. 1, Additional file 1). A control experiment with dasatinib alone at a dose of 1 mg/kg or 10 mg/kg, without *E. coli,* showed that dasatinib itself did not cause alveolar protein leak or increased cellular influx to the alveolar space (Supplementary Fig. 2, Additional File 1). All subsequent in vivo experiments examined outcome measures 24 h after intratracheal (*i.t.)* instillation of 1 × 10^6^ colony forming units (cfu) *E. coli*.

**Fig. 1:**
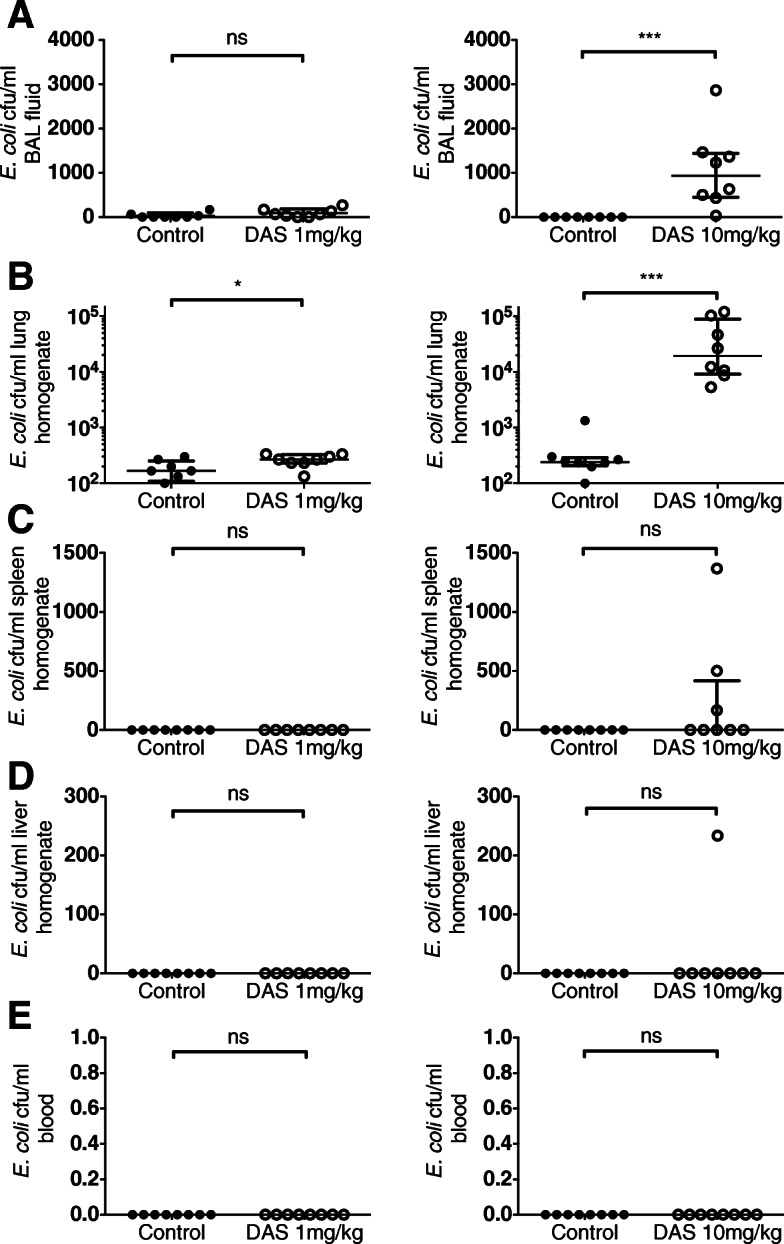
Effect of dasatinib pre-treatment on *E. coli* presence and survival in **a**, BAL fluid, **b**, lung homogenates, **c**, spleen, **d**, liver and **e**, peripheral blood from mice exposed to *i.t. E. coli*. *p < 0.05, ***p < 0.001. Values were derived from two experiments (1 mg/kg or 10 mg/kg dasatinib vs. control administered at 0 and 12 h) using 8 mice per group. DAS = dasatinib

Intraperitoneal (*i.p.*) dasatinib treatment at 0 h and 12 h was associated with a dose-dependent increase in the concentration of bacteria retrieved from the lungs of mice after *i.t.* administration of *E. coli* (delivered 30 min after the first dasatanib treatment), as compared to *i.p.* vehicle control (Fig. 1). Extra-pulmonary isolation of *E. coli* was observed in a small number of mice treated with the higher dose of dasatinib, 10 mg/kg. (Fig. 1).

**Fig. 2:**
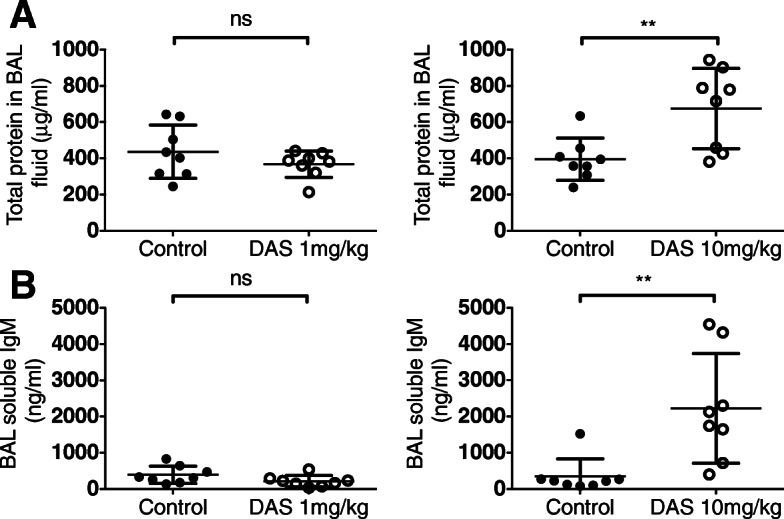
Effect of dasatinib pre-treatment on alveolar protein leak. **a** Total protein in BAL fluid. **b** Soluble IgM in BAL fluid from mice exposed to *i.t. E. coli*. **p < 0.01. Values were derived from two experiments (1 mg/kg or 10 mg/kg dasatinib vs. control administered at 0 and 12 h) using 8 mice per group

The increased pulmonary bacterial load observed after treatment with dasatinib at a dose of 10 mg/kg was associated with reduced barrier function of the alveolar-capillary membrane, as evidenced by significantly increased concentrations of total protein in bronchoalveolar lavage (BAL) fluid obtained 24 h after instillation of bacteria (Fig. 2a). This observation was verified by measuring IgM, as an example of a large molecule, the concentration of which is closely regulated in BAL fluid (Fig. 2b).

### Dasatinib has no effect on neutrophil recruitment or degranulation

**Fig. 3:**
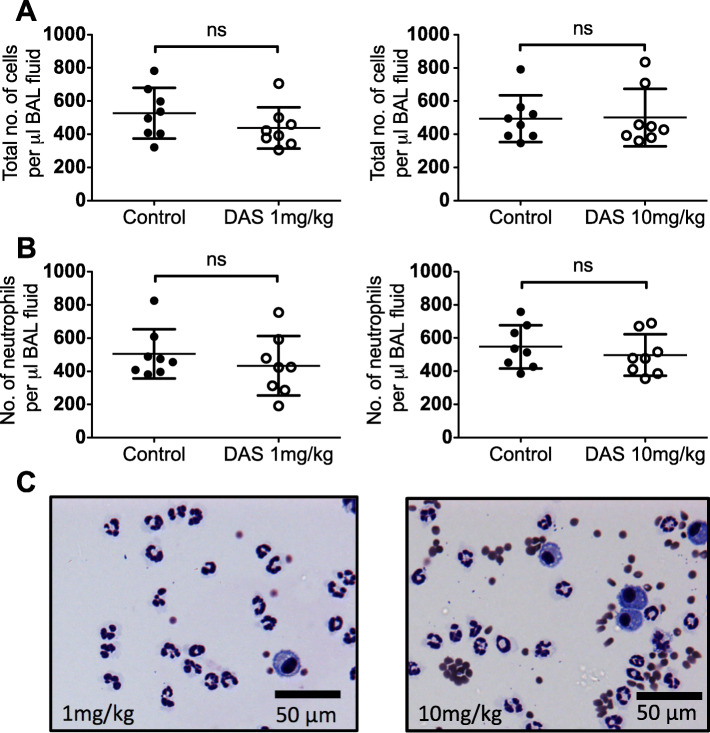
Effect of dasatinib pre-treatment on cellular influx into the alveolar space. **a** Total number of cells in BAL fluid. **b** Total number of neutrophils in BAL fluid. **c** Representative cytocentrifuge preparations of BAL fluid (400x magnification) from mice exposed to *i.t. E. coli*. Values were derived from two experiments (1 mg/kg or 10 mg/kg dasatinib vs. control administered at 0 and 12 h) using 8 mice per group

Disruption to the alveolar-capillary membrane can be caused by infiltration of activated leukocytes [9, 10, 19]. However, dasatinib had no influence on the proportion of leukocytes in BAL fluid, which was consistently ~ 85% leucocytes and ~ 15% alveolar macrophages in control, 1 mg/kg or 10 mg/kg dasatinib-treated mice, after *E. coli* treatment (Fig. 3). In keeping with this, concentrations of pro- and anti-inflammatory cytokines in BAL fluid were similar in the dasatinib and control groups, with small increments in mean levels of keratinocyte chemoattractant (KC) and tumour necrosis factor (TNF) in BAL fluid from mice treated with dasatinib at 10 mg/kg (see Supplementary Fig. 3, Additional file 1). No differences were observed in the number of pulmonary interstitial neutrophils in the dasatinib and control groups (see Supplementary Fig. 4, Additional file 1).

**Fig. 4:**
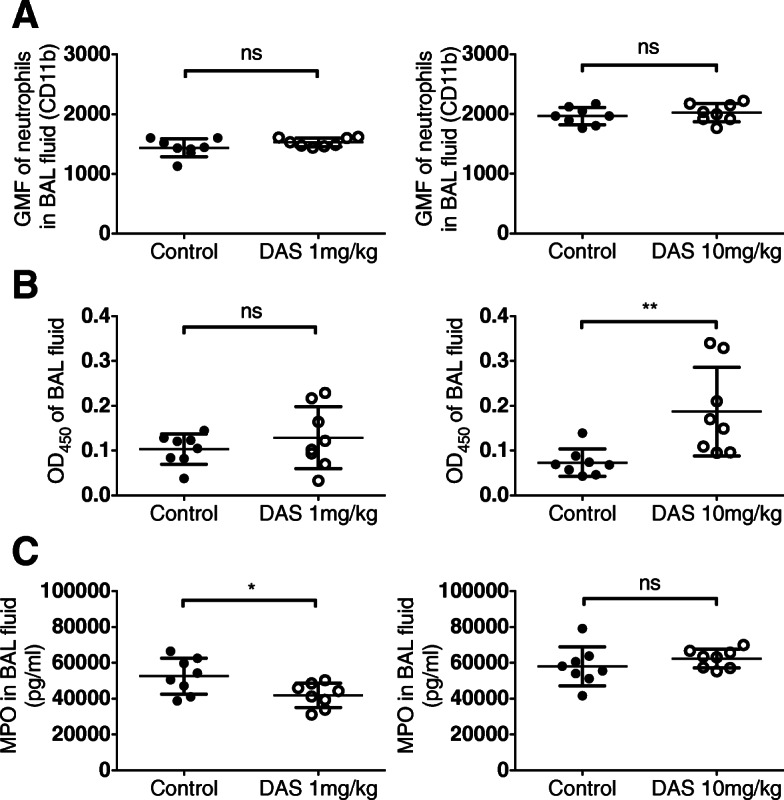
Effect of dasatinib pre-treatment on markers of neutrophil degranulation in the alveolar space. **a** CD11b expression on BAL neutrophils, **b** MPO activity and **c** MPO concentration in BAL fluid from mice exposed to *i.t. E. coli*. *p < 0.05, **p < 0.01. Values were derived from two experiments (1 mg/kg or 10 mg/kg dasatinib vs. control administered at 0 and 12 h) using 8 mice per group. GMF = geometric mean fluorescence

Dasatinib appeared to have a minimal effect on degranulation of neutrophils in vivo (Fig. 4). No difference in expression of neutrophil CD11b was found (Fig. 4a). Myeloperoxidase (MPO) activity was increased after treatment with dasatinib at a dose of 10 mg/kg, with no effect on concentrations of extracellular MPO antigen (Fig. 4b and c).

### Dasatinib increases extrapulmonary toxicity

Extrapulmonary toxicity also appeared to be increased by the higher concentration of dasatinib (10 mg/kg), which was associated with a consistent increase in plasma levels of the hepatic enzyme alanine transaminase (ALT), and a small but statistically significant increase in concentrations of plasma lactate (see Supplementary Fig. 5, Additional file 1).

### Dasatinib inhibits a number of key neutrophil functions in vitro

In an attempt to determine whether subtle effects on neutrophil function may contribute to the failure of bacterial clearance in vivo, human neutrophils isolated from peripheral blood were exposed to dasatinib in vitro. Experiments were carried out to determine whether dasatinib had any direct toxic effects on neutrophils. Dasatinib had no influence on release of lactate dehydrogenase (LDH), or on neutrophil apoptosis (see Supplementary Fig. 6, Additional file 1).

**Fig. 5:**
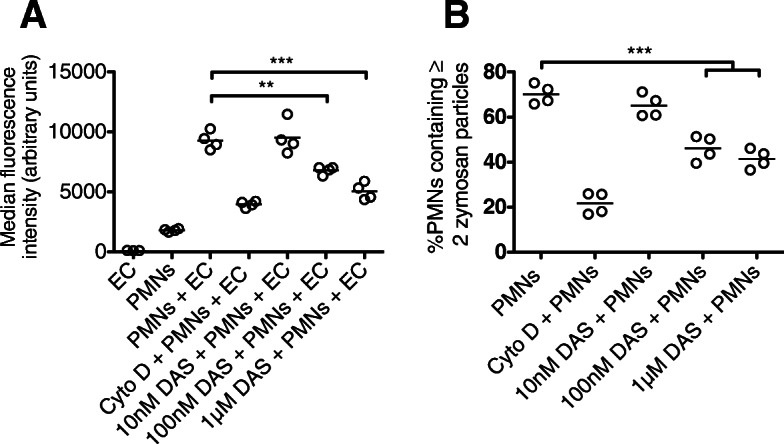
In vitro phagocytosis by isolated human blood neutrophils pre-treated with dasatinib. **a** MFI values of adherent neutrophils exposed to pHrodo *E. coli* particles (EC) following pre-treatment with cytochalasin D or dasatinib. **b** Dose response of dasatinib treatment on neutrophil phagocytosis of autologous serum-opsonized zymosan particles. ***p < 0.001. Values were derived from four experiments, each using neutrophils from different healthy volunteers

**Fig. 6:**
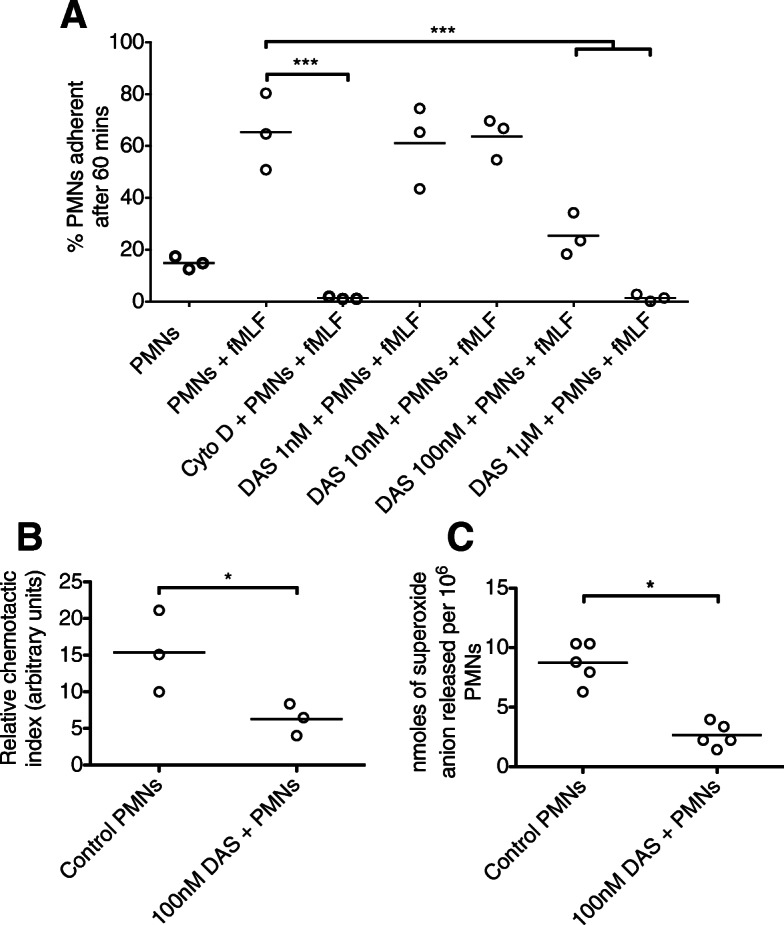
Effect of dasatinib on isolated human blood neutrophil adherence, chemotaxis and superoxide production in vitro. **a** Relative adherence of stimulated neutrophils to tissue culture plastic and **b** relative 2-D chemotactic index of neutrophils towards formylated peptide, following pre-treatment with dasatinib. **c** Extracellular superoxide production by stimulated neutrophils pre-treated with dasatinib. *p < 0.05, ***p < 0.001. Values were derived from three (A + B) or five (C) experiments, each using neutrophils from different healthy volunteers

**Fig. 7:**
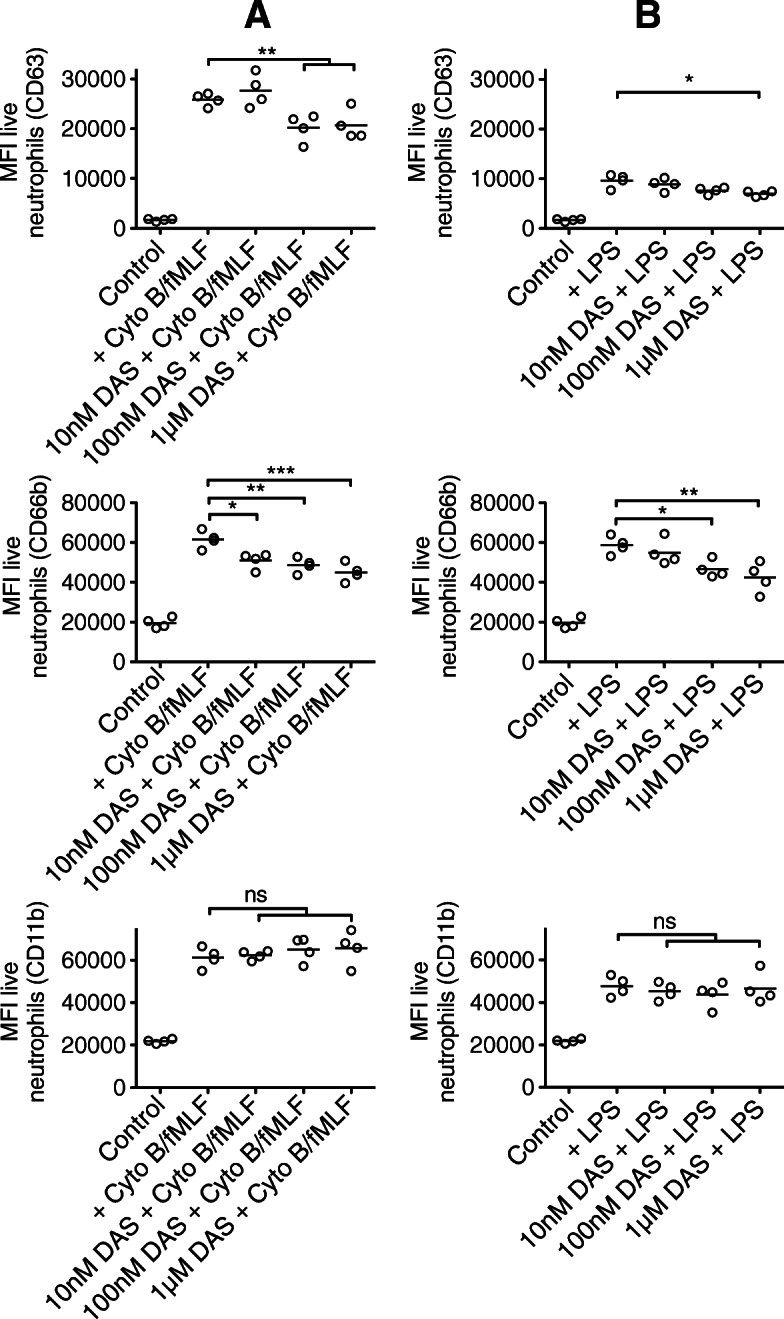
Effect of dasatinib on markers of degranulation on stimulated human neutrophils in heparinized blood. Flow cytometric analysis of CD63, CD66b and CD11b expression on gated neutrophil population following pre-treatment with dasatinib and stimulation with **a**, cytochalasin B + fMLF or **b**, LPS. *p < 0.05, **p < 0.01, ***p < 0.001. Values were derived from four experiments, each using neutrophils from different healthy volunteers

Dasatinib treatment resulted in significant impairment in the capacity of neutrophils to phagocytose *E. coli* particles or zymosan (Fig. 5). Dasatinib was also associated with significant reductions in neutrophil adhesion, chemotaxis and superoxide anion generation (Fig. 6). Dasatinib had small absolute effects on neutrophil degranulation (including that induced by *E. coli* LPS), though at higher concentrations of dasatinib, statistically significant inhibition of CD63 (primary granule) and CD66b (secondary granule) was observed (Fig. 7).

## Discussion

Our data indicate that src kinase inhibition with dasatinib is associated with dose-dependent impaired clearance of *E. coli* in vivo and in vitro. To our knowledge this is the first in vivo experimental demonstration modeling an increased susceptibility to pulmonary infection (with associated impairment of organ function) induced by dasatinib.

Our findings suggest that the failure to clear *E. coli* from the mouse lung is not attributable to impaired neutrophil recruitment, as the numbers of neutrophils entering the mouse lung were not significantly impaired by dasatinib. The in vitro experiments however suggested that neutrophil chemotaxis toward fMLF and adhesion to tissue culture plastic is inhibited by dasatinib. Several explanations could underlie the apparent discrepancy between the in vivo and in vitro findings – for example, the range of chemotactic signals in vivo is likely to be far broader and more complex. Furthermore, the stimulus strength of live bacteria may be too potent for the doses of dasatinib reaching the lung in situ to inhibit chemotaxis, adhesion and migration significantly. Ultimately we feel that an effect of dasatinib on neutrophil recruitment and migration seems unlikely to explain the observed impairment of clearance of *E. coli*. These data are in contrast to the beneficial effects of src kinase inhibition with dasatinib in the CLP sepsis model, where the lower dose of 1 mg/kg dasatinib administered 30 min before, 6 h and 24 h after CLP (compared with our own time course of 30 min before and 12 h after *i.t. E. coli*), improved survival and sepsis severity, as well as reduced intraperitoneal bacterial growth and spread [18]. Possible explanations for these differences are the timings of dasatinib administration after the acute inflammatory stimulus in the CLP model and the differing stimulus intensity between the two models. Similar to our findings, at the higher dose of 10 mg/kg dasatinib, there was a harmful effect in the CLP model.

We believe that the in vitro inhibitory effects of dasatinib on degranulation, superoxide generation and chemotaxis are not borne out in our in vivo model of infection, where the more likely explanation for impaired bacterial clearance may relate to inhibitory effects of dasatinib on phagocytosis. Dasatinib impaired ingestion of *E. coli*, and also inhibited phagocytosis of zymosan particles in vitro. The possibility remains that phagocytosis in vivo may have been further inhibited by the impairment in neutrophil adhesion induced by dasatinib, which has also been described by others [20–22]. Phagocytosis is more efficient when neutrophils are adherent to a substratum [23] and the implication, at least in vitro, is that dasatinib impairs tethering of neutrophils to surfaces. In contrast to dasatinib’s inhibition of phagocytosis, we did not observe the hypothesized marked effect on neutrophil degranulation.

In addition to suggesting a potential link to increased infection risk in patients prescribed dasatinib, our results provide an important caveat to recent studies suggesting a potential therapeutic role for src kinase inhibitors in inflammatory lung disorders. For example, src kinase inhibitors including dasatanib have been shown to significantly reduce lung injury and inflammation in animal models of acute lung injury [17, 24–26]. Similarly, beneficial anti-inflammatory effects have been attributed to dasatinib in experimental models of allergic asthma and acute silicosis [27, 28]. The acute lung injury studies described were all notable for using chemical and/or physical stimuli (rather than infective stimuli) to produce lung damage. Our findings extend the existing literature by suggesting that src kinase inhibitors deserve further study as anti-inflammatory medications, while keeping in mind that they may exacerbate infective causes of lung inflammation, and increase the risk of secondary infection.

We believe there are several strengths to this study. These included the use of doses of dasatinib used in comparable studies in the literature, and the reproducibility of in vivo data (as reflected by similar results for our separate control groups in Figs. 1, 2, 3), which in turn increases confidence in the observed effects of dasatinib. Furthermore, we used a broad range of assays interrogating different human neutrophil functions, with a view to understanding potential relevance in the human setting.

We also recognize that there were limitations in our study. A key consideration is whether our findings are compatible with existing literature. In particular, the elegant study by Gonçalves-de-Albuquerque et al. showed that dasatinib reduced lung infection in a CLP model [18]. There were fundamental differences between the studies including strain of mouse, route of administration of dasatinib (gavage versus our *i.p.* route) and the model (peritoneal, polymicrobial infection with secondary lung infection versus our direct, primary lung infection). The direct model consistently yields lung parenchymal infection and milder systemic change, and we opted for *i.p*. administration to avoid first-pass metabolism effects. However, we acknowledge that gavage more closely mimics the oral administration in patients, and that our data cannot exclude a beneficial effect of dasatinib in lung infection secondary to severe, systemic sepsis. Our findings should therefore be considered in the specific context of acute, primary pneumonia.

Secondly, it is possible that dasatinib has subtly different effects on innate immunity in mice and in humans [29, 30]. Our in vitro data in human neutrophils show that dasatinib inhibits degranulation from primary and secondary granules at higher concentrations, but clear evidence for a similar effect in mice was lacking. Also, pneumonia in patients is very rarely induced by a sudden bolus of infective material, as is traditionally used in mouse models of pneumonia. Furthermore, we have investigated one potential pathogen here. While noting these important limitations, we would defend the current study on the grounds that, as described above, a large prospective study of whether dasatinib is associated with pneumonia during use in patients would be extremely difficult to fund and perform at present, and no good rodent model imitating the true pathogenesis of human pneumonia is currently available to our knowledge. As such, we believed it important to perform a thorough assessment of one clearly relevant organism. We therefore conclude that our study has demonstrated proof of principle for in vivo susceptibility to pulmonary infection associated with dasatinib (and established conditions to explore this area further), but that these limitations must be kept in mind.

Third, it is extremely difficult to know whether the doses and concentrations of dasatanib used here are representative of concentrations of dasatinib in patients’ lung tissue. Prospective clinical studies specifically seeking to determine whether dasatanib increases the risk of pneumonia in patients would require prohibitively large numbers of subjects, and sub-studies to determine tissue concentrations of dasatinib in the lung of infected patients would be extremely challenging. Certainly, no such studies could be funded without supportive preliminary data demonstrating proof of principle for a pro-infective role of dasatanib in vivo (which we believe is provided here). Therefore, for this initial study, we selected doses used in comparable in vitro and in vivo studies.

Fourth, we acknowledge that studies of src kinase inhibitors are necessarily complicated by variations in tyrosine kinase inhibitor specificity. Dasatinib has inhibitory effects on abl kinases in addition to src kinases [31, 32]. The spectrum of src kinase inhibition for dasatinib differs from that of other src kinase inhibitors used in cancer research, such as PP1 or PP2. More specific individual src family kinase inhibitors e.g. for *Hck* and Fgr are not currently available, but would theoretically represent a potential way of selectively controlling individual neutrophil granule subtype activity to maintain phagocytosis and killing functions, while attenuating harmful extracellular degranulation [33]. However we selected dasatinib precisely because of its clinical relevance. As such we consider dasatinib to be the appropriate agent for this study.

## Conclusions

In summary, src kinase inhibition with dasatinib inhibits clearance of *E. coli* from the mouse lung, without influencing neutrophil recruitment. Instead, dasatinib impairs the capacity of neutrophils to adhere and to phagocytose *E. coli*. These data provide novel experimental evidence suggesting that src kinase inhibition is unlikely to provide an effective treatment strategy for infective acute lung injury.

## Methods

### In vivo acute lung infection model

C57BL/6 (female aged 8–12 weeks old; Charles River) received 1 mg/kg or 10 mg/kg dasatinib (Cambridge Bioscience, Cambridge, UK) *i.p*. in dimethyl sulfoxide or vehicle control. After 30 min, animals received *i.t. E. coli* (serotype ATCC25922, grown to log phase) at a dose of 1 × 10^6^ cfu as described [34]. Dasatinib or vehicle control were re-administered *i.p.* at 12 h and samples obtained at 24 h.

### Assessment of bacterial load

At 24 h, BAL was performed with three aliquots of 800 μl sterile ice-cold saline administered *i.t*., and sequentially gently aspirated. 50 μl BAL from the first lavage was kept for flow cytometric analysis of neutrophils and bacterial determination, with the remainder centrifuged at 300 *g* for 5 min, with cell-free supernatant from the first lavage stored at -80 °C for subsequent biochemical analysis. Blood samples were taken by direct cardiac puncture and added to citrate (as anticoagulant). Following lavage lungs were perfused with 20 ml phosphate-buffered saline and removed en bloc prior to digestion using 5 mg/ml collagenase D (Roche) in HBSS with cations [34]. Blood, BAL fluid and homogenized lung, liver and spleen samples were all incubated overnight at 37 °C on LB agar plates to determine bacterial colony counts.

### Quantification of neutrophils

After collagenase digestion of the right lung, red blood cells were lysed (ACK lysis buffer, Invitrogen) and Fc-mediated antibody binding reduced by incubation with Fc block (Mouse BD Fc block, BD Bioscience). Lung digest cells, BAL fluid cells and whole blood were incubated with anti-CD45 and anti-Ly6G antibodies (Biolegend, London, UK) for 30 min at 4 °C in the dark. After addition of FACSlyse (BD Bioscience) cells were resuspended in PBS and 50 μl flow-check fluorospheres added (Beckman Coulter, Brea, CA) prior to flow cytometric analysis.

### Assays of neutrophil degranulation in vivo

BAL fluid cell samples were incubated with anti-Ly6G, anti-CD63 and anti-CD11b antibodies (Biolegend, London, UK), with flow analysis of neutrophil populations. CD63 is a membrane protein expressed on azurophilic (primary) granules and CD11b/CD18 (Mac-1) is a β2 integrin expressed across specific (secondary), gelatinase (tertiary) and secretory vesicles [35]. Both can be used as surrogate markers of degranulation when these granules fuse with the extracellular membrane [36]. BAL fluid MPO activity was quantified using the tetramethylbenzidine (TMB)/hydrogen peroxide (H_2_O_2_) colorimetric assay [37], while MPO concentration was quantified using a mouse MPO enzyme-linked immunosorbent assay (ELISA) kit (R&D Systems, Abingdon, UK).

### Quantification of BAL cytokines and alveolar protein concentration

The pro- and anti-inflammatory cytokine profile of BAL fluid was quantified using ELISA kits for KC, monocyte chemoattractant protein-1 (MCP-1), TNF and interleukin (IL)-10 (R&D Systems, Abingdon, UK) according to the manufacturers’ instructions. Total protein was measured using a Pierce bicinchoninic acid assay kit (Life Technologies, Paisley, UK) and BAL fluid IgM by ELISA (eBioscience, Hatfield, UK).

### Quantification of systemic toxicity

Plasma alanine transaminase (ALT) and lactate were quantified according to manufacturers’ instructions (Alpha Laboratories, Eastleigh, UK and Randox Laboratories, Crumlin, UK respectively) and adapted for use on a Cobas Fara centrifugal analyzer (Roche Diagnostics, Burgess Hill, UK).

### Isolation of neutrophils from human blood

Neutrophils were isolated from the citrated whole blood of healthy donors by dextran (Pharmacosmos, Holbaek, Denmark) sedimentation and fractionation through isotonic discontinuous Percoll (GE Healthcare, Little Chalfont, UK) gradients, as described [38, 39]. Neutrophils were re-suspended at assay-dependent concentrations in warmed Iscove’s modified Dulbecco’s medium (IMDM, Life Technologies, Paisley, UK) with 1% autologous serum. Only preparations yielding > 95% neutrophil purity with > 95% viability were used in experiments.

### Neutrophil phagocytosis assay

Human neutrophils adherent to tissue culture plastic were exposed to autologous serum-opsonized pH sensitive pHrodo™ green *E. coli* Bioparticles for 30 min at 37 °C (Life Technologies, Paisley, UK), before analyzing fluorescence at 585 nm.

In separate assays, neutrophils were exposed for 30 min at 37 °C to zymosan particles (derived from *Saccharomyces cerevesiae*) that had been opsonized using autologous serum. Phagocytosis of zymosan particles was quantified as described [40].

### Neutrophil adhesion assay

Neutrophil adhesion to plastic was adapted from previously described methods [41]. Briefly, fresh neutrophils in IMDM were added to a 24-well plate with 50 nM fMLF (Sigma, Gillingham, UK) for 60 min to promote adhesion. Non-adherent cells were washed off before adherent cells were trypsinized, suspended with CountBright™ Absolute Counting Beads (Life Technologies, Paisley, UK) and counted by flow cytometric analysis.

### Neutrophil chemotaxis assay

Two-dimensional ‘under agarose’ chemotaxis towards fMLF was measured as described [42].

### Neutrophil superoxide assay

The extracellular release of superoxide anion by neutrophils in response to priming by platelet-activating factor (PAF, Sigma, Gillingham, UK) and activation by fMLF was quantified using the cytochrome C reduction assay [43].

### In vitro neutrophil degranulation assays

Fresh heparinized blood samples were stimulated with 10 μM cytochalasin B (Sigma, Gillingham, UK)/1 μM fMLF or *E. coli* 026:B6*-*derived LPS (Sigma, Gillingham, UK) to induce neutrophil degranulation before incubating with anti-CD63, anti-CD66b (localized on secondary neutrophil granules) and anti-CD11b antibodies for flow analysis, as described [36].

### Neutrophil toxicity assays

Direct cytotoxicity of dasatinib on isolated neutrophils was quantified using a Roche LDH cytotoxicity kit (Roche, Sussex, UK), according to the manufacturer’s instructions. Cytocentrifuge preparations of isolated neutrophils exposed to dasatinib for 24 h and stained with Giemsa (Sigma, Gillingham, UK) were assessed for morphological changes consistent with apoptosis.

### Statistical analysis

Flow cytometry data were analyzed using Flojo version 10.0.7 (Ashland, OR, US). Results, expressed as individual data points with mean ± s.d. (in vivo data) or mean (in vitro data), were analyzed using GraphPad Prism (Graphpad Software, La Jolla, CA, US), by repeated measures one-way analysis of variance or a Friedman test with a post-hoc Dunn’s test, or an unpaired t-test, as appropriate. Statistical significance was considered to be at the level of *p* < 0.05.

## Supplementary information

**Additional file 1 **Intratracheal instillation of *E. coli* resulted in the anticipated time-dependent accumulation of neutrophils in the pulmonary interstitium and alveolar space, with marked inflammation present at 24 h, and near resolution by 48 h (Supplementary Fig. 1). Dasatinib alone, without *i.t. E. coli,* caused no cellular influx into the alveolar space or alveolar protein leak (Supplementary Fig. 2). When *E. coli*-infected mice were treated with either dasatinib or control at 0 and 12 h, small mean increments were seen in BAL fluid concentrations of the pro-inflammatory cytokines KC and TNF in association with dasatinib at 10 mg/kg (Supplementary Fig. 3), but no differences were observed in the numbers of neutrophils in the pulmonary interstitium (Supplementary Fig. 4). A small increase in blood neutrophils was observed in association with the higher dose of dasatinib studied (10 mg/kg) (Supplementary Fig. 4). Treatment of *E. coli*-infected mice resulted in a significant increase in blood levels of lactate and of the hepatic enzyme ALT (Supplementary Fig. 5). When dasatinib or control was applied directly to isolated human blood neutrophils, no difference was observed in the release of LDH or on the rate of neutrophil apoptosis (Supplementary Fig. 6). **Figure S1** Kinetics of neutrophil influx into the lung interstitium and alveolar space following *i.t.* instillation of *E. coli* in mice. *n* = 2–3 mice per time point. **Figure S2** Effect of dasatinib pre-treatment alone on cellular influx to the alveolar space and alveolar protein leak. *n* = 1 mouse per condition. **Figure S3** Effect of dasatinib pre-treatment on the pro/anti-inflammatory cytokine profile of the alveolar space. **A,** KC, **B,** MCP-1, **C,** TNF and **D,** IL-10 concentrations in BAL fluid from mice exposed to *i.t. E. coli*. *p < 0.05, **p < 0.01, ****p < 0.0001. Values were derived from two experiments (1 mg/kg or 10 mg/kg dasatinib vs. control administered at 0 and 12 h) using 8 mice per group. **Figure S4** Effect of dasatinib pre-treatment on blood neutrophilia and neutrophil influx into lung interstitium. **A,** % neutrophils of all leukocytes in blood and **B,** total number of neutrophils in lung homogenates from mice exposed to *i.t. E. coli*. *p < 0.05. Values were derived from two experiments (1 mg/kg or 10 mg/kg dasatinib vs. control administered at 0 and 12 h) using 8 mice per group. **Figure S5** Effect of dasatinib pre-treatment on markers of extrapulmonary toxicity. **A,** plasma lactate and **B,** plasma ALT concentrations in mice exposed to i.t. *E. coli*. **p < 0.01, ****p < 0.0001. Values were derived from two experiments (1 mg/kg or 10 mg/kg dasatinib vs. control administered at 0 and 12 h) using 8 mice per group. **Figure S6** Effect of dasatinib on the viability and apoptosis of isolated human blood neutrophils. **A,** cytotoxicity of dasatinib, as measured by extracellular LDH release, expressed as a percentage of the sample lysed using Triton. **B,** apoptosis rates after 24 h of culture with PMA (phorbol myristate acetate) or dasatinib, as measured by cell morphological characteristics under light microscopy. **p < 0.01. Values were derived from three (A) or four (B) experiments, each using neutrophils from different healthy volunteers.

## Data Availability

The datasets used and/or analysed during the current study are available from the corresponding author on reasonable request.
