# Supplementary material for: Src kinase inhibition with dasatinib impairs neutrophil function and clearance of Escherichia coli infection in a murine model of acute lung injury
Source: J Inflamm (Lond). 2020 Oct 30;17:34. doi: 10.1186/s12950-020-00261-5 (PMC7597020; doi:10.1186/s12950-020-00261-5)
Supplement: Supplementary file 1 — Additional file 1 Intratracheal instillation of E. coli resulted in the anticipated time-dependent accumulation of neutrophils in the pulmonary interstitium and alveolar space, with marked inflammation present at 24 h, and near resolution by 48 h (Supplementary Fig. 1). Dasatinib alone, without i.t. E. coli, caused no cellular influx into the alveolar space or alveolar protein leak (Supplementary Fig. 2). When E. coli-infected mice were treated with either dasatinib or control at 0 and 12 h, small mean increments were seen in BAL fluid concentrations of the pro-inflammatory cytokines KC and TNF in association with dasatinib at 10 mg/kg (Supplementary Fig. 3), but no differences were observed in the numbers of neutrophils in the pulmonary interstitium (Supplementary Fig. 4). A small increase in blood neutrophils was observed in association with the higher dose of dasatinib studied (10 mg/kg) (Supplementary Fig. 4). Treatment of E. coli-infected mice resulted in a significant increase in blood levels of lactate and of the hepatic enzyme ALT (Supplementary Fig. 5). When dasatinib or control was applied directly to isolated human blood neutrophils, no difference was observed in the release of LDH or on the rate of neutrophil apoptosis (Supplementary Fig. 6). Figure S1 Kinetics of neutrophil influx into the lung interstitium and alveolar space following i.t. instillation of E. coli in mice. n = 2–3 mice per time point. Figure S2 Effect of dasatinib pre-treatment alone on cellular influx to the alveolar space and alveolar protein leak. n = 1 mouse per condition. Figure S3 Effect of dasatinib pre-treatment on the pro/anti-inflammatory cytokine profile of the alveolar space. A, KC, B, MCP-1, C, TNF and D, IL-10 concentrations in BAL fluid from mice exposed to i.t. E. coli. *p < 0.05, **p < 0.01, ****p < 0.0001. Values were derived from two experiments (1 mg/kg or 10 mg/kg dasatinib vs. control administered at 0 and 12 h) using 8 mice per group. Figure S4 Effect of dasatinib [file 12950_2020_261_MOESM1_ESM.pptx]

## Slide 1
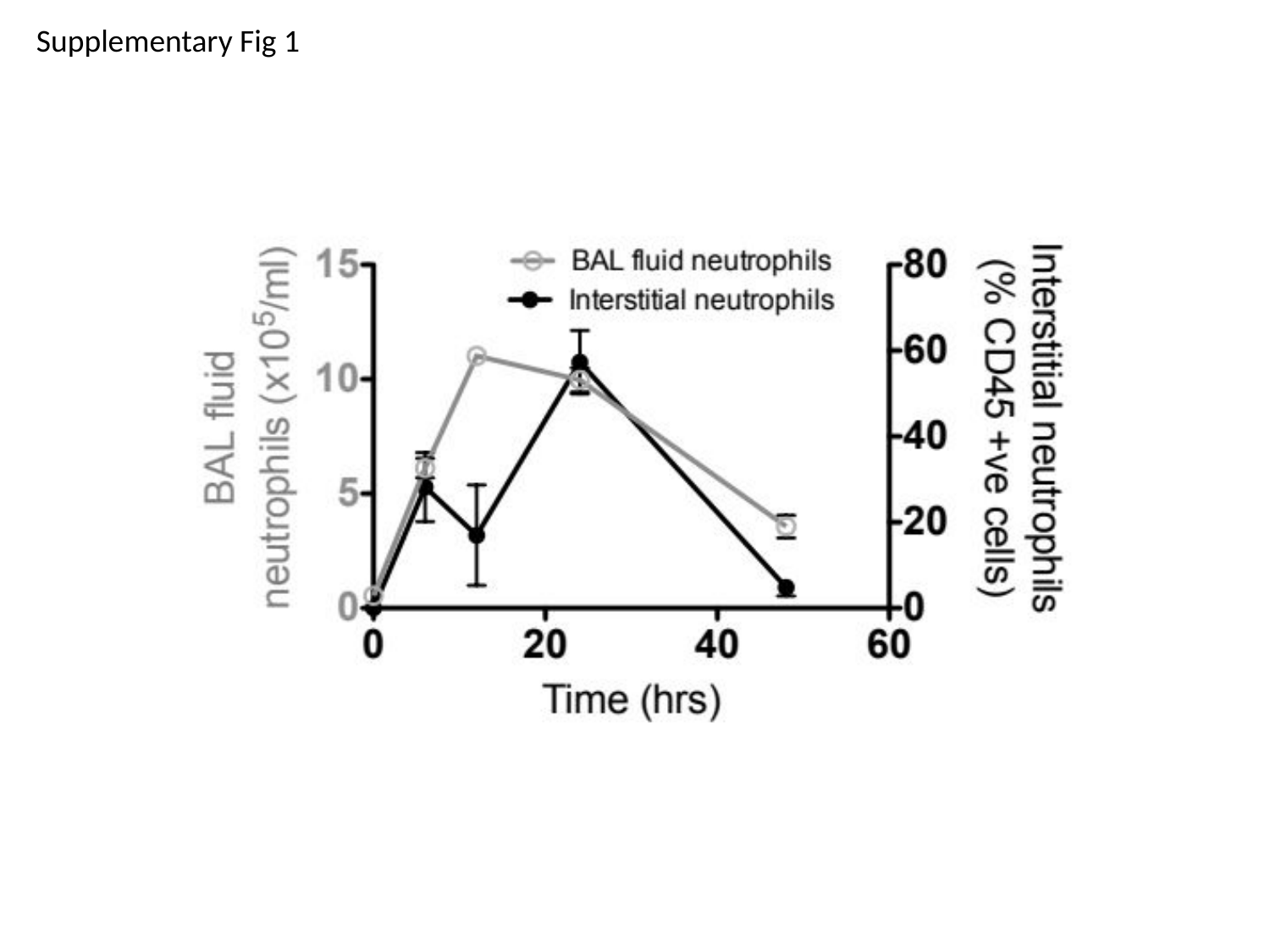

Supplementary Fig 1

## Slide 2
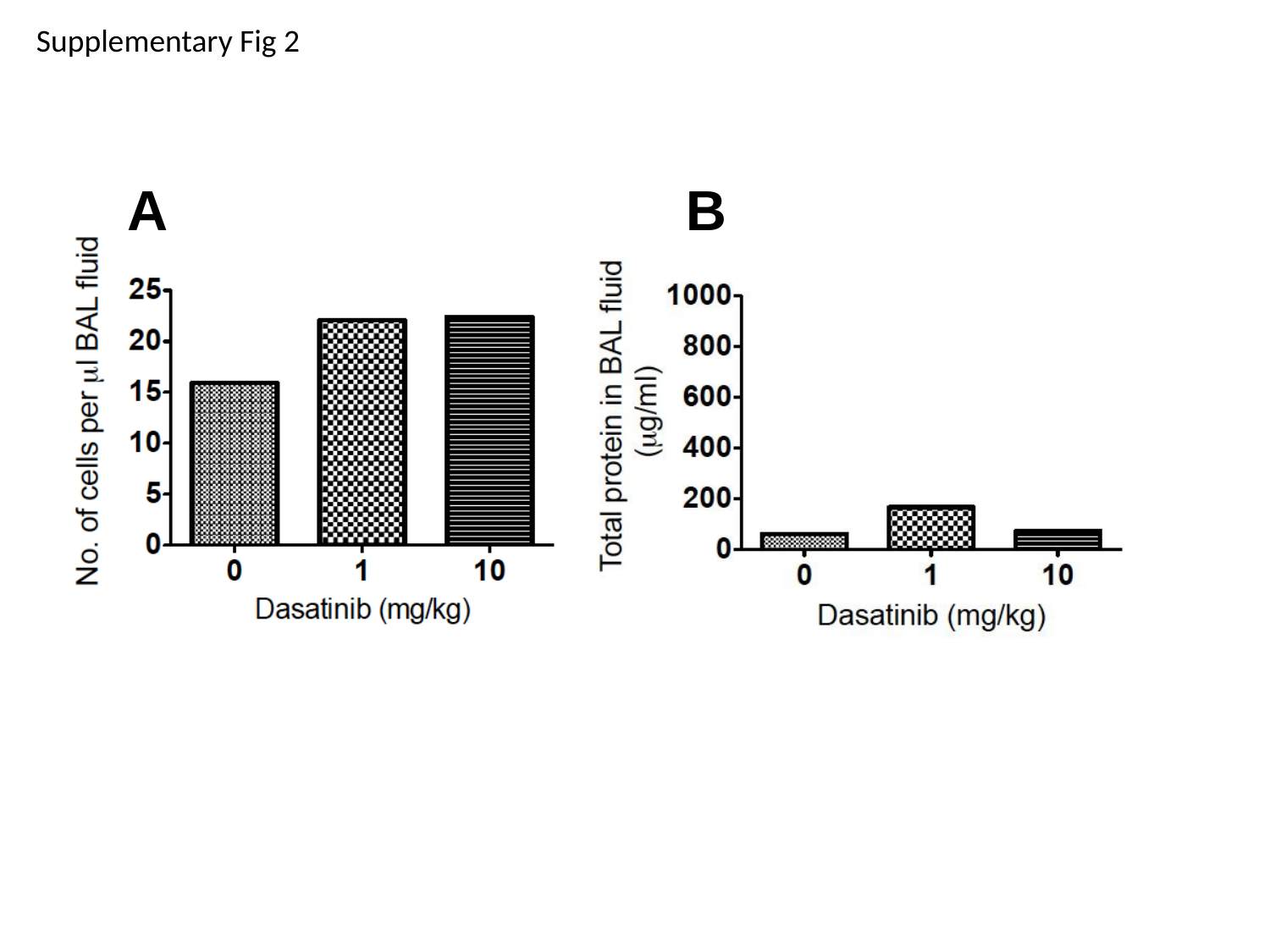

Supplementary Fig 2
A
B

## Slide 3
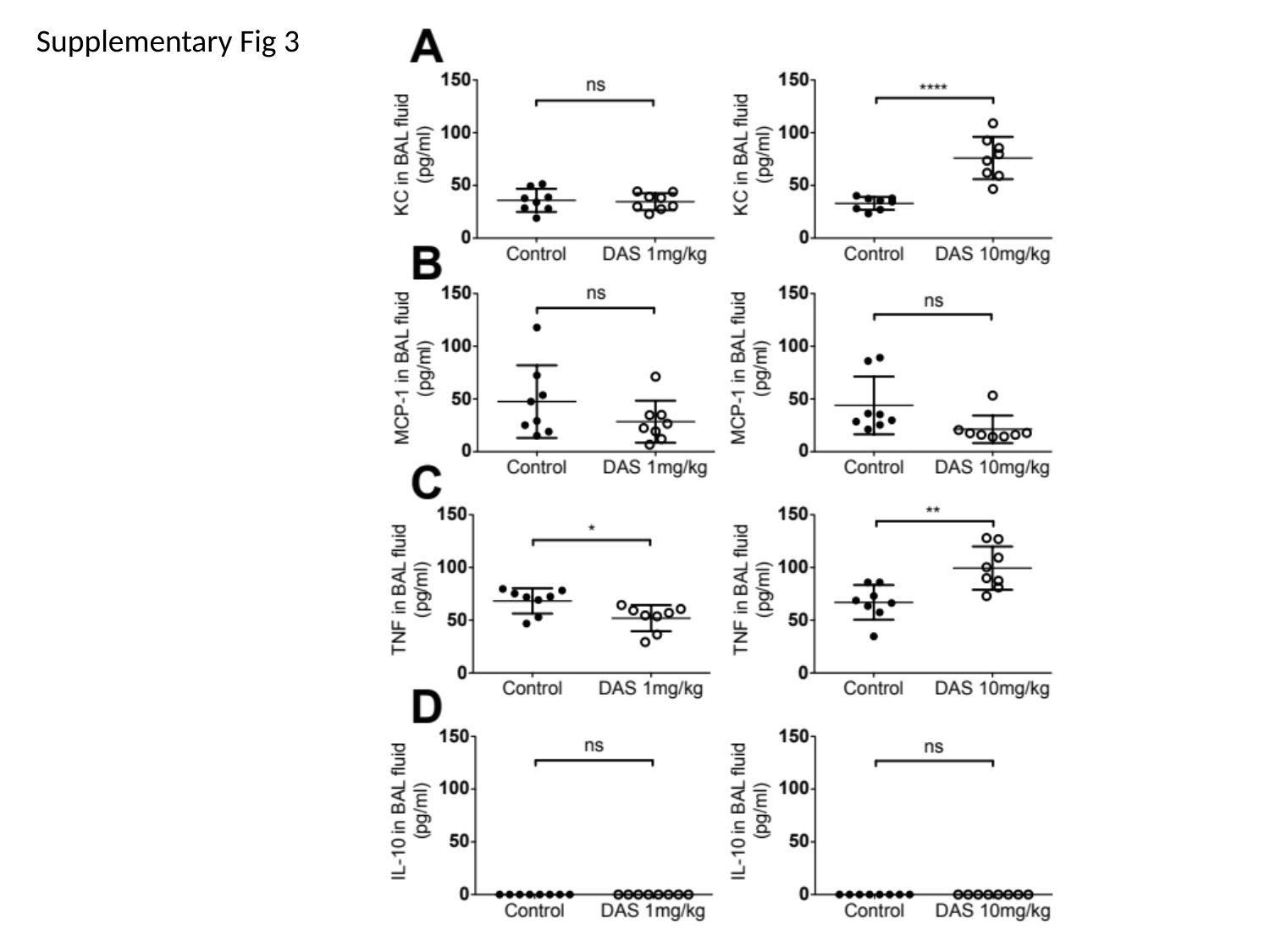

Supplementary Fig 3

## Slide 4
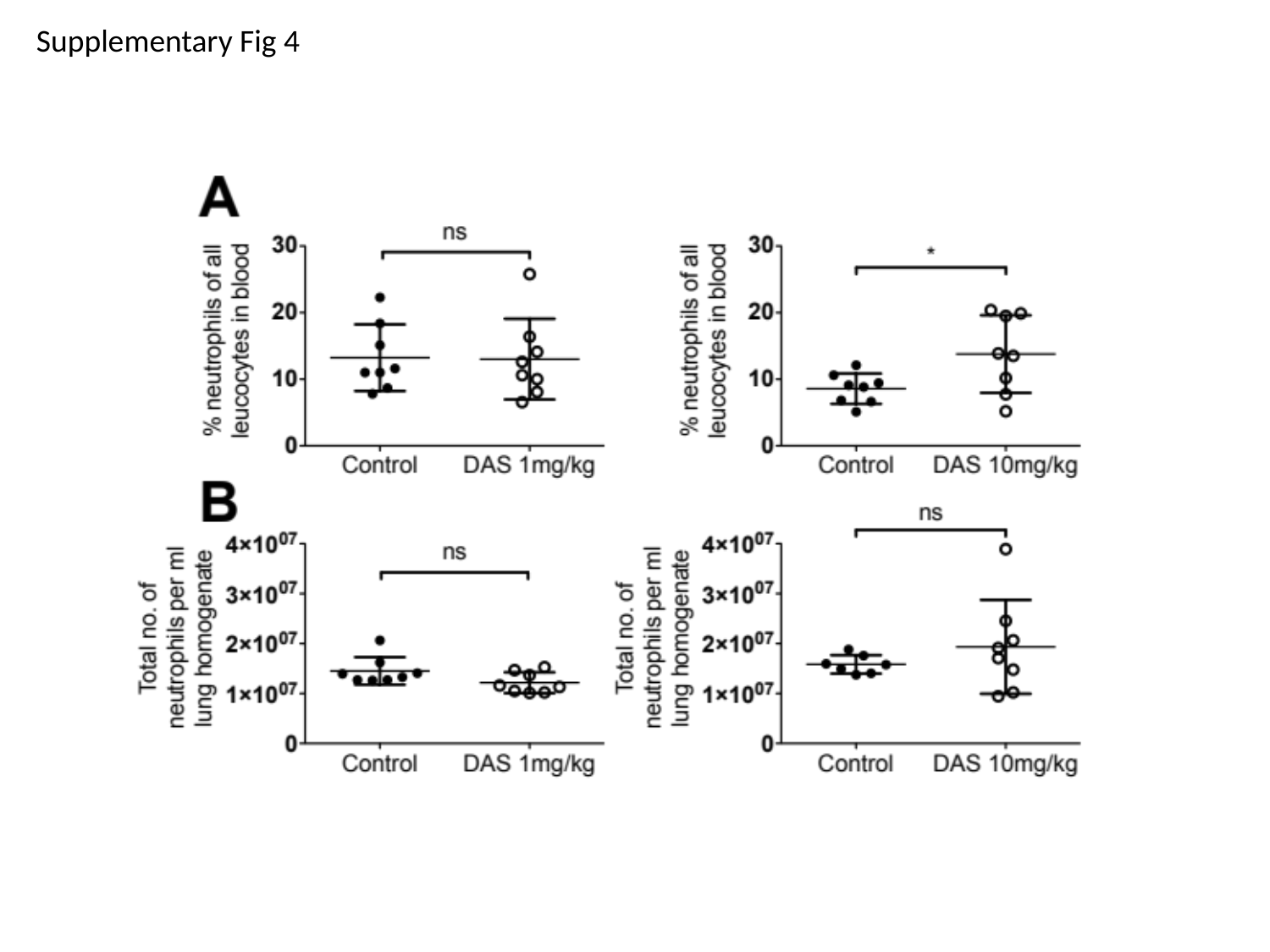

Supplementary Fig 4

## Slide 5
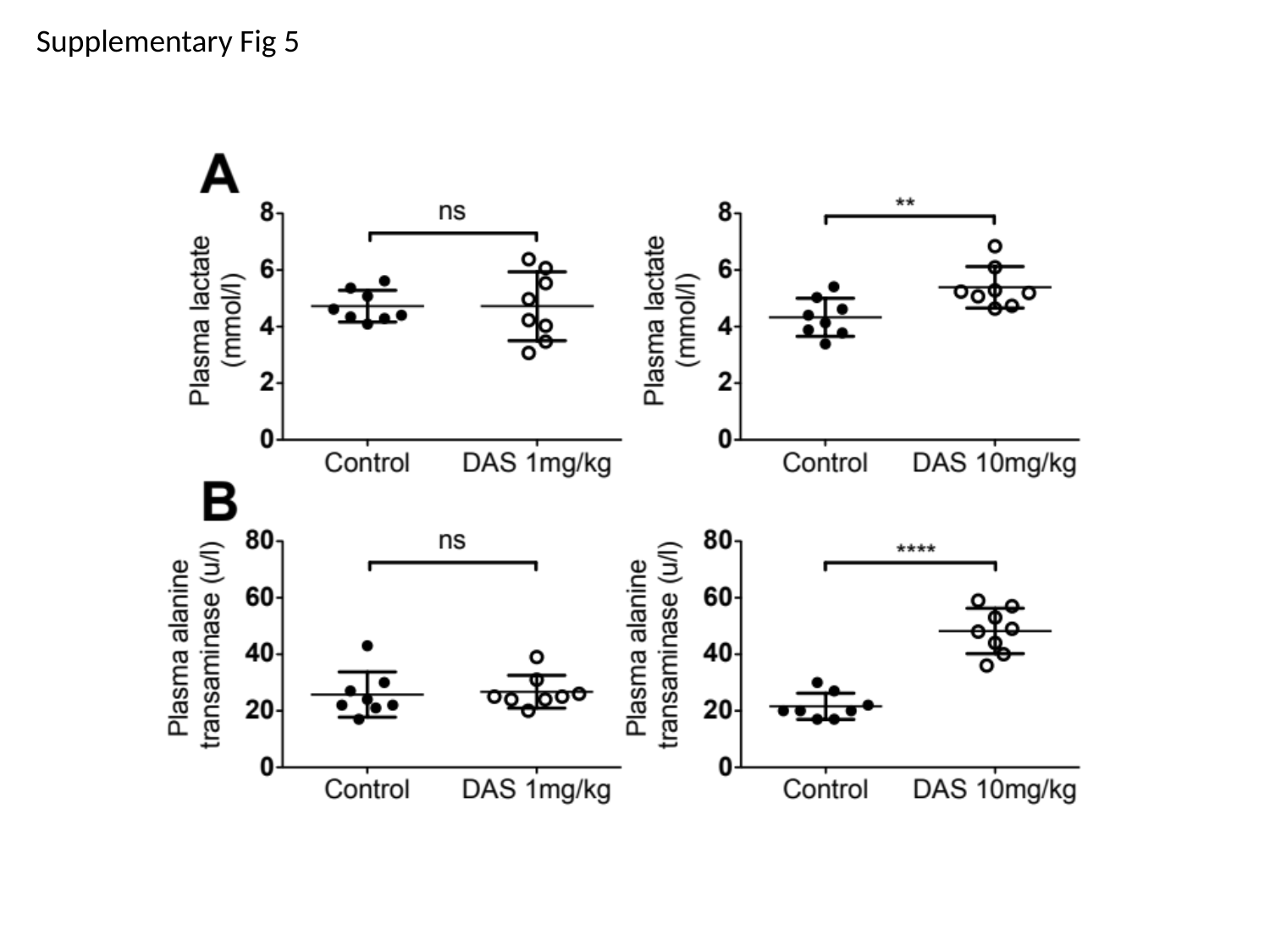

Supplementary Fig 5

## Slide 6
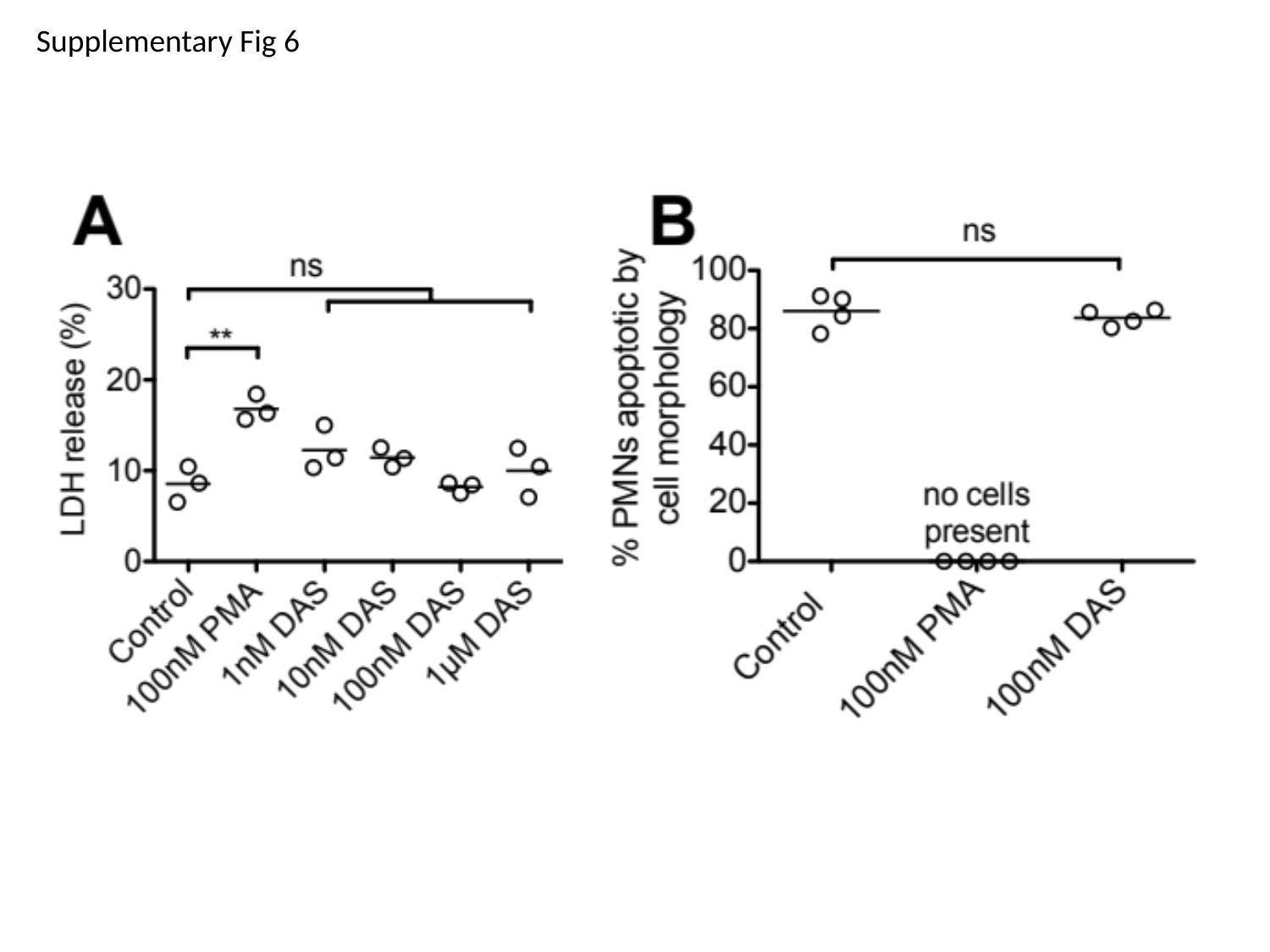

Supplementary Fig 6
